# Comparison of Four Different Dental Implant Removal Techniques in Terms of the Weight and Volume of Bone Loss

**DOI:** 10.7759/cureus.61104

**Published:** 2024-05-26

**Authors:** Nuthaphat Chittavoravanich, Bundhit Jirajariyavej, Sompop Bencharit, Prakan Thanasrisuebwong

**Affiliations:** 1 Implant Dentistry, Faculty of Dentistry, Mahidol University, Bangkok, THA; 2 Prosthodontics, Faculty of Dentistry, Mahidol University, Bangkok, THA; 3 Workman School of Dental Medicine, High Point University, High Point, USA

**Keywords:** piezosurgery, bur, trephine drill, counter torque ratchet, implant removal

## Abstract

Purpose: Several approaches have been suggested for implant removal. However, further research is necessary to review data regarding the amount of bone removed and the duration of removal time for different procedures. This study evaluates and compares various implant removal techniques.

Materials and methods: A polyurethane block was scanned to create an implant surgical guide. Afterward, implant-guided surgery was performed on 60 simulated bone blocks. The implants were then separated into four groups and removed utilizing the counter-torque ratchet, trephine drills, burs, and piezosurgery.

Results: For the weight of bone loss, there were significant differences in the median between the counter-torque ratchet technique (CTRT) and trephine (p < 0.01), CTRT and bur (p < 0.01), trephine and piezo (p < 0.01), and bur and piezo (p = 0.04). All groups, except CTRT and the piezo group, demonstrated a statistically significant difference (p < 0.01) in the procedure durations. Regarding the volume of bone loss, a statistically significant difference (p < 0.01) was found between each group.

Conclusions: CTRT showed the least amount of bone loss. On the other hand, the trephine technique was demonstrated to be the fastest. It is essential to consider the limitations and risks when choosing the approach for implant removal.

## Introduction

Dental implants serve as a reliable treatment option for both edentulous and partially edentulous patients seeking to replace missing teeth, restore function, and enhance their appearance [1-5]. Despite their high success rate, complications arise in approximately 33.6% of implants within the first five years, leading to implant failures [6-8]. The etiological explanations for dental implant failures can be categorized into biological failures (peri-implantitis, failure to attain or maintain osseointegration), mechanical failures (implant fracture), iatrogenic failures (bone overheating, implant malposition, site contamination), and functional failures (prosthesis design, functional overload) [7,9]. Implant removal, particularly in cases without significant peri-implant bone loss, poses challenges, and traumatic removal may hinder immediate implant placement, complicating prosthetic rehabilitation [10]. Hence, precise and conservative removal of an osseointegrated implant is crucial, considering factors such as the amount of peri-implant residual bone, proximity to vital anatomical structures, and implant design. Careful treatment planning can mitigate treatment duration, side effects, and costs [11], with a focus on preserving peri-implant bone for immediate or future implant placement [12].

Various implant removal techniques have been proposed, including the counter-torque ratchet technique (CTRT), trephine drills, burs, piezosurgery, and laser surgery, either individually or in combinations, each offering distinct benefits, limitations, and overall success rates [13]. While reverse torque is considered conservative for peri-implant tissues, allowing potential immediate implant insertion, it may not be suitable for fractured implants, narrow implants (under 4 mm), or specific implant systems. Trephine drills, despite being a fast and successful treatment, pose invasiveness concerns in free-hand trephine implant removal, potentially damaging peri-implant tissues and reducing success rates for immediate implant placement. Caution is advised, especially when using the free-hand trephine method with guided approaches. The burs technique, effective in removing peri-implant bone, may require a lengthy clinical time, leaving challenging bone defects for immediate implant placement. Additionally, bur removal may result in implant or bur materials in the wound, interfering with proper healing. Piezosurgical devices, minimally invasive to hard tissues, can be time-consuming for implant removal. Laser surgery, minimally invasive like piezosurgery, requires longer operational time compared to conventional methods, and further research is needed for laser-assisted implant removal [14]. Roy et al.'s systematic review identified five techniques with varying success rates: reverse torque (87.7%), trephines (100%), burs (94%), piezosurgery (100%), and erbium, chromium-doped yttrium, scandium, gallium and garnet (Er,Cr:YSGG) laser (100%), concluding that despite its lower success rate, reverse torque is the most conservative and should be the primary choice for implant removal [15].

The critical nature of the implant removal method lies in its potential impact on future implant placement and prosthetic rehabilitation. Damage to hard and soft tissues, bone overheating, and the presence of contaminated implant or instrument material particles can adversely affect healing time, the number of surgeries, and restoration fabrication time, influencing the patient's financial and overall well-being [16]. Therefore, the choice of implant removal techniques should align with clinical considerations such as implant site, alveolar bone quantity and quality, and site-specific anatomical characteristics. During the planning of implant removal, careful consideration of limits and potential complications is essential [17]. However, there remains a need for additional research to investigate data on the amount of bone removed and implant removal time for different techniques. The study's goal is to evaluate and compare various implant removal techniques, including CTRT, trephine drills, burs, and piezosurgery, focusing on bone removal quantity and operational time.

## Materials and methods

This comparative study was conducted at the Dental Implant Center, Faculty of Dentistry, Mahidol University, Bangkok, Thailand, from February 2023 to December 2023.

Surgical guide fabrication

**Figure 1:**
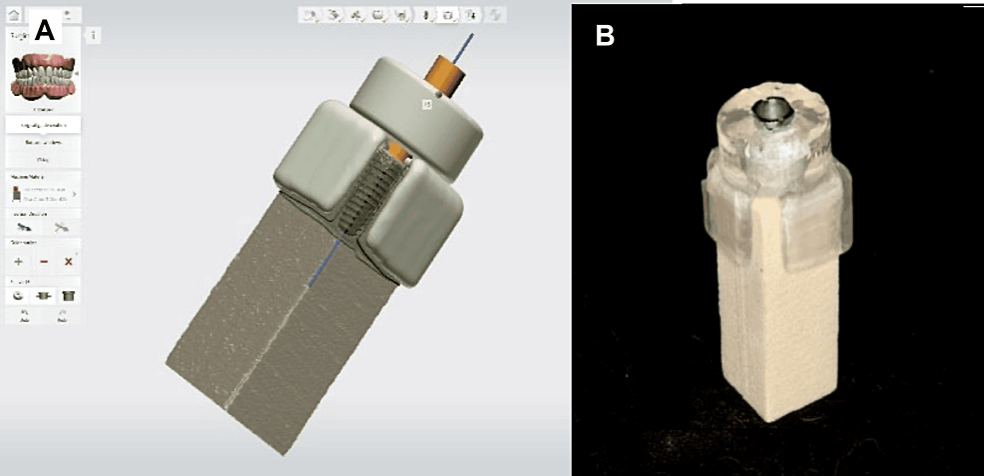
Implant guide design (A) and final specimen (B)

A simulated polyurethane bone block measured 15 x 15 x 40 mm in size was used to mimic the physical properties of the cortical and cancellous bones. The block was scanned using a laboratory 3D scanner (Ceramill Map 600; Amann Girrbach AG, Vorarlberg, Austria) to generate the standard tessellation language (STL) file that was used to create a customized implant surgical guide using 3Shape Implant Studio (3Shape, Copenhagen, Denmark). A 4.5 × 11.5 mm implant (IS-II active; Neobiotech Co. Ltd, Suwon, South Korea) was placed into the block using guided implant surgery protocol per the manufacturer’s recommendation (Figure 1). The implant's coronal location was set to be 0 mm deep (crestal level) in the center of the block. The surgical guide parameters were set for a thickness of 2.00 mm, model offset of 0.0200 mm, and a 10.5 mm sleeve offset specific to Neobiotech SGS70RW (Neobiotech Co. Ltd). Two inspection windows were incorporated to ensure proper fitting of the surgical guides on the block. Subsequently, the guide was produced using a 3D printer (Form 3B; Formlabs Inc., Somerville, Massachusetts, United States). 

Implant placement procedure

**Figure 2:**
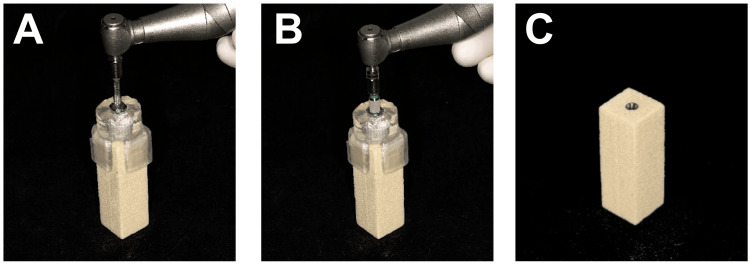
Surgical placement of an implant into a simulated bone block (A) Implant guided surgery; (B) Implant insertion; (C) Simulated bone block after placed with implant.

Prior to the procedure, a 3D surgical guide was applied to the bone block, and the stability and fit were evaluated through windows and tactile inspection. Sixty dental implants (IS-II active, Neobiotech Co. Ltd) were placed using guided surgery into 60 simulated bone blocks. All implants were weighed on a laboratory digital weighing scale (Analytical Balance AL54; Mettler Toledo, Columbus, Ohio, United States) before the experiment to establish the baseline weight. Implant osteotomy sites were prepared following a fully guided surgical protocol (Neo NaviGuide Kit; Neobiotech Co. Ltd), adhering to the manufacturer's recommended sequence of surgical drills to accommodate a 4.5×11.5 mm implant. Implants were inserted into each bone block using a surgical handpiece with an insertion torque of 50 Ncm, and all implant platforms were positioned at the crestal bone level (Figure 2). All implants were placed by a single well-trained implant surgeon. Following this, the implants were divided into four groups based on distinct implant removal procedures.

Implant removal protocol

All implant removal was performed using common clinical protocols as well as the manufacturer's instructions. The removal procedure was applied until the implant reached a clinically loose state, then forceps were utilized for final implant retrieval. A proficient implant surgeon, skilled in both implant insertion and removal procedures, conducted all aspects of the surgical operations. All implant removal was performed by a single well-trained implant surgeon.

CTRT

**Figure 3:**
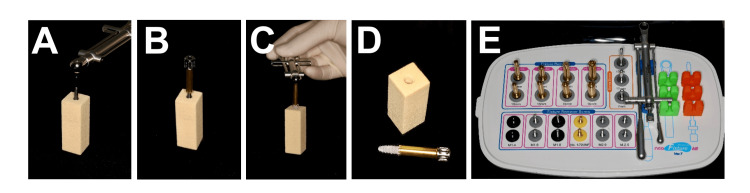
Implant removal using counter-torque ratchet technique (A) The fixture removal screw was connected to the implant and used FR screw hex driver to apply torque to the implant; (B) The fixture remover was inserted in an anti-clockwise orientation onto the removal screw; (C) The torque control device was applied in an anti-clockwise manner until the implant rotate; (D) Simulated bone block after the implant was removed; (E) The fixture removal kit.

The fixture removal kit (Neobiotech Co. Ltd) was employed using the CTRT (Figure 3). The procedure commenced by selecting an appropriate implant fixture removal screw for the implant's screw access (in this study, FRS18, M1.8). Subsequently, the implant fixture removal screw was inserted into the implant fixture screw access. The next step involved connecting the implant fixture removal screw to the torque drive (in this study, HDF1612, 12 mm) and ratchet. The fixture remover screw was then securely installed in a clockwise direction with a torque ranging between 40 and 80 Ncm (with a maximal setting of 40 Ncm utilized in this study). Following this, the fixture remover (in this study, Wide (Ø5), FR520, 20mm) connected to the fixture remover screw was slowly countered to torque the implant in a counterclockwise orientation. Finally, the torque control device was applied with counterclockwise force for implant removal until the implant became loose and was successfully retrieved.

Burs Technique

**Figure 4:**
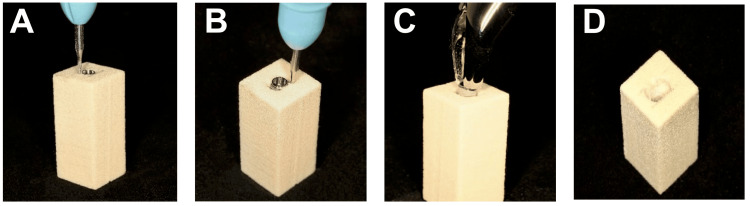
Implant removal using burs technique (A) A fissure carbide bur was utilized to remove bone; (B) Bone material was removed mesially and distally from the implant; (C) The implant was grabbed with surgical forceps and rotated both clockwise and anti-clockwise; (D) Simulated bone block after the implant was removed.

For the bur technique, a fissure carbide bur (C21.HP.012; Jota AG, Rüthi, Switzerland), measuring 1.2 mm in diameter and 4.1 mm in length, was utilized at a speed of 6,000 rpm, as recommended by the manufacturer (Figure 4). The procedure commences with marking the block at the buccal and lingual contours to establish a reference line, restricting removal exclusively to the mesial and distal sides of the implant. Subsequently, bone material was removed mesially and distally from the implant, directed towards the apex, maintaining a depth of 4.1 mm while closely approaching the implant surfaces. The implant head was then gripped with surgical forceps (150 Upper Universal, IS Forceps; Hu-Friedy, Chicago, Illinois, United States) and rotated both clockwise and anti-clockwise to preserve both the buccal and lingual bone. As resistance diminished, the implant was removed with a concluding anti-clockwise rotation.

Trephine Drill Technique

**Figure 5:**
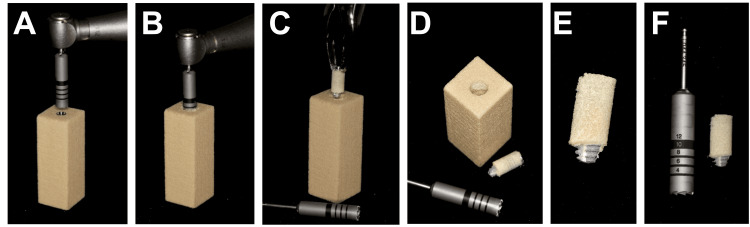
Implant removal using trephination (A) A trephine drill; (B) An osteotomy was made around the implant to a depth of 10 mm; (C) Surgical forceps were employed to remove the implant; (D) Simulated bone block after the implant was removed; (E) A bone ring surrounding the removed implant; (F) Trephine drill and removed implant.

A trephine drill (Explantation drill, medium, 4.9 mm; Straumann Group, Basel, Switzerland) with an inner diameter of 4.9 mm and an outer diameter of 5.5 mm was used (Figure 5). The trephine drill had an overall length of 37.5 mm and operated at a speed of 200 rpm, adhering to the manufacturer's instructions. The trephine was utilized to create an osteotomy around the implant by penetrating through the block to a depth of 10 mm, resulting in the loosening of the implant. Subsequently, surgical forceps (150 Upper Universal, IS Forceps; Hu-Friedy) were employed for the final removal of the implant.

Piezosurgery

**Figure 6:**
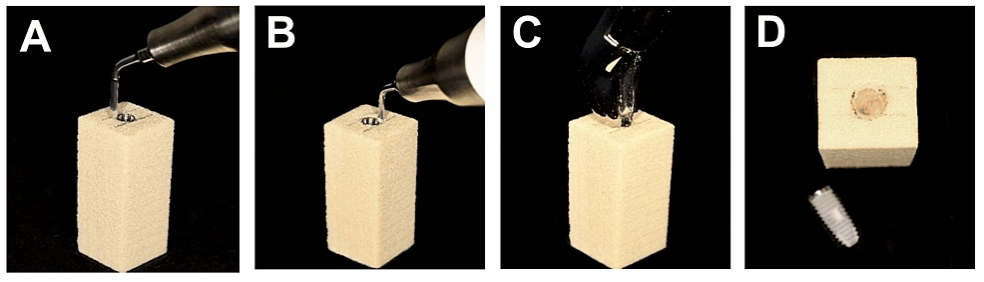
Implant removal using piezosurgery (a) Piezosurgery device with a surgical tip LC2; (b) An osteotomy was performed around the implant to a depth of 5 mm; (c) Surgical forceps were utilized to remove the implant; (d) Simulated bone block after the implant was removed.

Piezosurgery devices (Piezotome, Acteon Group, Norwich , England) were operated using a surgical tip LC2 (Acteon Group) with the mode of Piezotome D4 (Figure 6). To limit the osseous removal to the mesial and distal side of the implant, the block was first marked at the buccal and lingual contour for a reference line. Then, the surrounding osseous removal was performed from the implant both mesially and distally, aiming to reach the apex at a depth of 5 mm (half of the tip’s length) while staying in close proximity to the implant surfaces. Utilizing the surgical forceps, the implant head is grabbed and rotated clockwise and counterclockwise. After that, soft rocking motions are used. A last counterclockwise turn removes the implant when minimal resistance is noticed. 

Data collection

The Weight of Bone Loss Measurement

**Figure 7:**
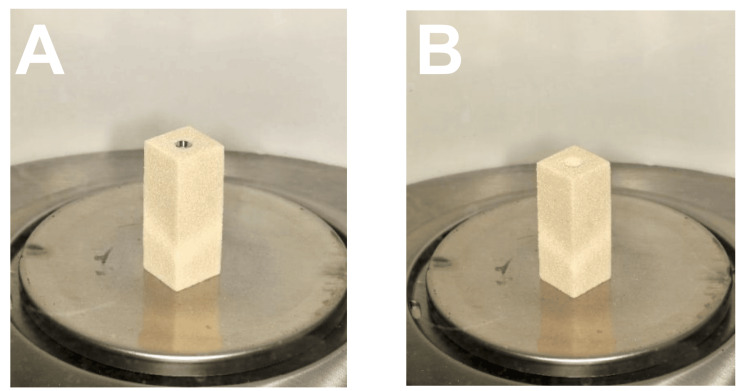
Weighting of the specimen before (A) and after (B) implant removal.

The loss of simulated bone material after implant removal was quantified in milligrams using a laboratory digital weighing scale (Analytical Balance AL54; Mettler Toledo). The reduction in bone quantity was determined by subtracting the weight of the bone block with the implant before and after implant removal (Figure 7). The material loss was calculated by subtracting the final combined weight of the block and implant from the original weight.

The Duration of Procedure Measurement 

The time taken to remove each implant was measured in seconds using a digital stopwatch (Casio Computer Co., Ltd., Shibuya, Tokyo, Japan). The procedure duration for each specimen was calculated by measuring the time after removing each implant individually.

The Volume of Bone Loss Measurement

**Figure 8:**
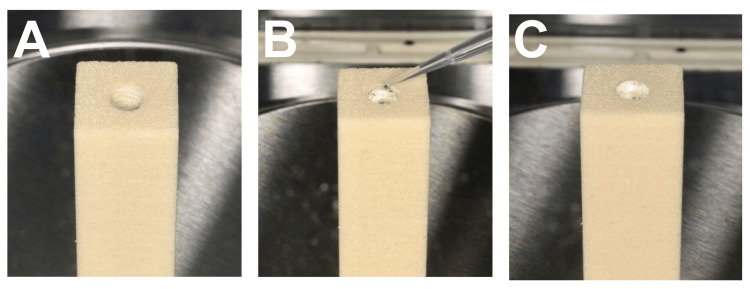
Water filling process to measure the volume of the implant removal site (A) The implant removal site before the process; (B) Deionized water was used to fill in the implant removal site using a micropipette; (C) The implant removal site was filled up to the crestal level of the block.

The volumetric measurement was done using deionized water (Ultra Clear® TP TWF Ultrapure Water Systems; Evoqua Water Technologies Pte Ltd., Pittsburgh, Pennsylvania, United States) to fill in the implant removal site using a micropipette (Mettler Toledo). Once the implant removal site was filled up to the crestal level of the block, the volume of the removed bone was identified by the increase in the total weight of the specimen. Deionized water was chosen for the experiment since water has a density of 1 g/mL, and the fluidity of the water allows it to flow into the implant removal site adequately after about one hour of filling the implant site based on the preliminary data. Using a laboratory digital weighing scale, the volume of bone loss following implant removal was determined in milligrams (Figure 8). 

Statistical analysis

IBM SPSS Statistics for Windows, Version 22.0 (Released 2013; IBM Corp., Armonk, New York, United States) was used to analyze all statistical data. The Shapiro-Wilk test was employed to validate the normality of the data. Since the data distribution was not normal, a non-parametric analysis, the Kruskal-Wallis test, was used to compare the differences in the weight of bone loss among the four different implant removal techniques. For differences in the duration of the procedures and volumetric bone loss in the four implant removal techniques, the data distribution was normal, and thus, the one-way ANOVA was applied. The statistical level of significance was set at 0.05.

## Results

Table 1 presents the median, percentile 25, and percentile 75 of the weight of bone loss among the four implant removal methods, while Table 2 displays the mean, SD, and p-values of the volume of bone loss and the duration of the procedures for the four implant removal techniques. 

**Table 1:** The differences in the weight of bone loss among the four implant removal techniques* *In the same measurement, different superscripts (a or b) within two groups indicate a statistically significant difference (p < 0.05). CTRT: counter-torque ratchet technique

	CTRT (n=15)		Trephrine (n=15)	Bur (n=15)		Piezosurgery (n=15)
Measurement		Median (Percentile25, Percentile75)				
Weight (g)	0.397 (0.395, 0.402)^a^		0.427 (0.426, 0.432)^b^	0.424 (0.418, 0.425)^b^		0.405 (0.403, 0.413)^a^

**Table 2:** The differences in the volume of bone loss and the duration of the procedures among the four implant removal techniques* *In the same measurement, different superscripts (a, b, c, or d) within two groups indicate a statistically significant difference (p < 0.05). Volume: ANOVA post-hoc Tukey multiple comparison test; Time: ANOVA post-hoc Games-Howell multiple comparison test CTRT: counter-torque ratchet technique

	CTRT (n=15)		Trephrine (n=15)	Bur (n=15)		Piezosurgery (n=15)
Volume (ml), mean± SD	0.1332 ± 0.0102^a^		0.2656 ± 0.0123^b^	0.2009 ± 0.0143^c^		0.1684 ± 0.0070^d^
Time (s), mean± SD	67.541 ± 3.691^a^		15.997 ± 1.093^b^	50.547 ± 3.218^c^		69.527 ± 5.119^a^

**Figure 9:**
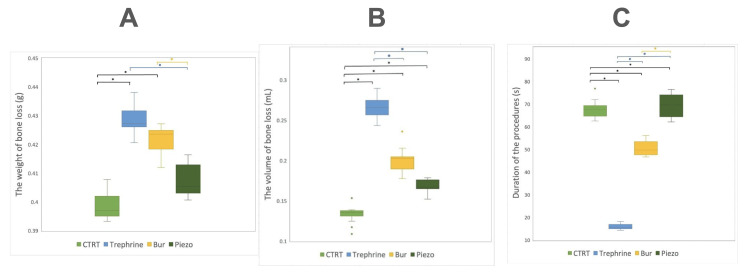
Boxplots demonstrating changes (A) the weight of the bone material removal; (B) volumetric bone loss; (C) operating time. The asterisk (*) indicates if a measurement difference between any two groups is statistically significant (p < 0.05).

Concerning the weight of bone loss, the trephine group exhibited the highest loss, followed by the bur, piezo, and CTRT groups, respectively. Statistical analysis (Figure 9) revealed significant differences in the median between CTRT and trephine (p < 0.01), CTRT and bur (p < 0.01), trephine and piezo (p < 0.01), and bur and piezo (p = 0.04).

Regarding the duration of the procedures, the mean duration of the trephine group was less than that of the other groups, with the piezo group having the longest duration. There was a statistically significant difference among all the groups (p < 0.01), except between CTRT and the piezo group. In terms of the volume of bone loss, the trephine group experienced the most considerable loss, followed by the bur, piezo, and CTRT groups, aligning with the differences observed in the weight of bone loss. A statistically significant difference (p < 0.01) was found between each group. The distribution of measurements is illustrated using boxplots (Figure 9).

## Discussion

This study investigated and compared various implant removal methods, including the counter-torque ratchet, trephine drills, burs, and piezosurgery, concerning the amount of bone removed and procedure time. Statistically significant differences were observed among all implant removal methods for the volume of bone loss and, between some groups for the weight of bone loss and duration of the procedures, the null hypothesis was rejected. While the vitro limitations of the study include the type of simulation bone model and a single implant surgeon, the results provide a true side-by-side comparison of all four most common clinical techniques of implant removal with a thorough examination of bone loss via weight and volume measurements. 

Based on the weight and volume of bone loss, the CTRT provided the least bone loss, followed by piezosurgery, bur, and trephine drill, respectively, in both measurements. Considering the duration of the procedures, trephine is the fastest method, whereas piezosurgery is the slowest one. The CTRT had the least amount of bone removed during implant removal. This is explained by the way this technique engages the implant and reverses it out of the bone using a torque applied counterclockwise. Minimal bone loss results from shear stress application because it breaks the contact between the implant and bone interface [9,13]. This method is perhaps one of the most conservative methods, requiring little or no bone removal and causing minimal harm to the surrounding tissues [15]. As a result, the CTRT alone or combined is suggested as the first alternative for removing failed non-mobile dental implants since it is a less invasive procedure [9]. This technique should be used only if the implant connection is intact and the implant is solid enough to be 'torqued out' without breakage. Implants with higher osseointegration should be removed using a combination of CTRT and a bone-cutting approach, such as trephine burs or piezoelectric bone cutters [9,13]. 

In this study, approximately 50 Ncm torque was applied for implant insertion, and for CTRT, approximately 60-70 Ncm was necessary for implant removal. Anitua et al. examined 42 patients who had 91 implants in total. Only a counter-torque ratchet was used to remove 78 implants, whereas 13 implants required a combination of trephine burs and the BTI device (BTI Biotechnology Institute, Álava, Spain) [12]. A study by Lee investigated 81 patients with 139 implants and discovered that 139 implants could be unscrewed with a torque of 146 Ncm [18]. A trephine bur was only necessary when the torque was greater than 200 Ncm. They showed success rates of 86% and 88% for implant removal using this technique. In another recent study by Anitua et al., 150 patients with 199 implants needing explanted were treated using a CTRT employing a reverse torque implant removal kit (Neo Fixture Remover; Neobiotech Co. Ltd) [17]. This technique had a 97 percent success rate, with 193 implants removed without flap elevation; the average removal torque was 220 Ncm. The only complication was an implant fracture while using the removal kit and being unable to remove two implants even with a maximum torque (500 Ncm). The investigators concluded that the CTRT could protect both hard and soft tissues, thus allowing the installation of a replacement implant later or immediately. They also suggested that the device might be a first choice for the elderly since it has a high success rate and is less intrusive, resulting in less postoperative morbidity and healing time. 

Trephination can be a traumatic procedure that causes considerable bone destruction surrounding the implant. To limit collateral injury to surrounding tissue, the trephine's internal diameter should be slightly bigger than the implant to avoid contacting the implant body and remove as little of the peri-implant bone as possible. When removing the surrounding bone of the implant to the coronal half, elevators or forceps can be used to remove the implant or as part of a combination approach. However, this technique is more aggressive than the reverse torque technique [13]. Despite its being more invasive for soft and hard tissues, trephine osteotomies are straightforward and fast to perform. Some implant manufacturers include guiding cylinders and sleeves for proper drill angulation. When using this procedure without a surgical guide, this is shown to be unpredictable since it is problematic to follow the axis of the implant, which might be critical with narrow ridges [11] The trephine bur is a better option when a fractured implant or abutment screw needs to be removed or when an implant is too firmly integrated with the bone [18-20]. Trephine burs have been related to various complications, including fatigue, fractures of the jaw, and osteomyelitis [18-20]. Hence, this procedure should only be done when alternative and less invasive procedures are not applicable. An in vitro experiment investigated the trephine method and compared it to the Er,Cr:YSGG laser regarding bone removal and procedure duration. The results showed that the quantity of bone removed when removing the implants was less in the laser group than in the trephine group, and the trephine burs, in terms of process time, was more than twice as fast as the laser. In conclusion, as compared to trephine burs, laser surgery was less invasive but was more time-consuming [21,22].

Burs were used in a few studies to remove osseointegrated implants, and the techniques were varied. Monje et al. suggested that the high-speed (20,000 rpm) or low-speed (800-1000 rpm) rotary burs are effective in removing bone and the cortical part in the event of initial failure of less invasive; on the other hand, this method is invasive, time-consuming, and challenging to perform immediate implant placement [14]. To avoid damaging the buccal plate of bone, cuts should be done on the mesial and distal sides of the implant, which is frequently enough to loosen the implant and remove it by the counter-torque ratchet approach [13]. This technique allows implant replacement with a similar size and design [23]. However, some implants required simultaneous bone grafts for immediate implant placement [23,24]. Stajčić et al. suggested two bur techniques for the explantation of dental implants; they concluded that when other approaches fail, these two bur techniques can be suggested as a safe and reliable alternative strategy [10]. 

Piezoelectric ultrasound utilizes ultrasound tips to oscillate and vibrate, effectively dividing solid surfaces like bone tissue. The ultrasonic vibrations allow for selective cutting in mineralized structures without causing harm to soft tissue. While this instrument offers improved surgical visibility and safety due to minor bleeding during deep cutting into bone, it may take longer to perform surgery compared to other methods [23-29]. Piezoelectric devices present a viable alternative to traditional osseous surgery, demonstrating high precision and safety in osteotomies without jeopardizing nearby soft tissues or essential structures [23-29]. Notably, piezo tips cause less trauma to surrounding soft tissue during peri-implant bone removal than high-speed rotary burs, and they exhibit a superior bone healing response compared to the bur technique in osteotomy preparation sites. Despite the extended duration of surgery, piezosurgery allows for conservative osteotomies and facilitates simultaneous implant placement in the same surgical site [23-29]. Research comparing osteotomies and osteoplasty using a carbide drill, a diamond drill, and piezosurgery found that piezosurgery technology promotes bone gain, distinguishing itself from carbide and diamond drills that cause bone loss. This underscores piezosurgery's notable capacity for bone regeneration and efficacy [25-29]. Messina et al. demonstrated successful implant removal using a piezosurgical device in 10 healthy patients, emphasizing accurate cutting with minimal bone damage, vascular structure protection, and hemostasis facilitation. The technique often enables simultaneous implant placement in the same surgical site, and there are no absolute contraindications reported in the literature for using piezoelectric devices in implant removal [30].

Clinical recommendations by Solderer et al. underscore the importance of comprehensive implant removal planning, considering factors such as implant stability, the osseointegrated interface, bone quality, available explantation techniques, invasiveness of protocols, and potential risks [8]. They advocate for the CTRT, either alone or in combination, as the preferred option to preserve alveolar bone. Roy et al. suggest selecting explantation methods based on cortical bone thickness, adjacent structures, and the timing of future implant placement [14]. Despite its lower success rate, the reverse torque approach is recommended as the initial choice, with alternative methods utilized if reverse torque fails. A piezoelectric device can be used in case the reverse torque exceeds 200 Ncm without success, and trephine drills or burs can be employed at low speeds if a piezoelectric device is unavailable. If the counter-torque technique fails or if the implant is fractured, bone removal may be completed using piezosurgery or burs. Trephine drills are recommended for bone-level implants with sufficient surrounding bone and space, with the use of guiding cylinders or guided surgery [15]. In summary, the authors strongly recommend using the reverse torque method for implant removal, employing conservative and careful approaches if needed in case of failure. Future clinical studies comparing these removal methods with other technologies such as guided surgery and lasers should be considered.

The choice of implant removal method should be guided by clinical parameters, including the integrity of the implant-abutment connection, implant location, remaining bone quantity and quality, and specific anatomical circumstances of the implant. Before implant removal, a treatment strategy and post-removal planning must be developed, thoroughly reviewed, and discussed with the patient. It is crucial to consider and communicate the limitations and potential risks associated with each selected procedure when deciding on the appropriate implant removal technique [13].

## Conclusions

The CTRT exhibited the least bone loss in contrast to the bur, trephine, and piezosurgery methods. However, the Trephine method demonstrated superior speed compared to the others. When choosing implant removal techniques, it is essential to assess the potential risks and limitations of each method, taking into account the reason for implant removal and relevant anatomical factors. A personalized approach that combines different methods for real-life patients should also be considered.
